# Comparative analysis of selected innate immune-related genes following infection of immortal DF-1 cells with highly pathogenic (H5N1) and low pathogenic (H9N2) avian influenza viruses

**DOI:** 10.1007/s11262-014-1151-z

**Published:** 2015-01-04

**Authors:** Ai-ling Liu, Yu-feng Li, Wenbao Qi, Xiu-li Ma, Ke-xiang Yu, Bing Huang, Ming Liao, Feng Li, Jie Pan, Min-xun Song

**Affiliations:** 1 The Key Laboratory of Animal Resistance Biology of Shandong, College of Life Sciences, Shandong Normal University, 88, East Culture Road, Jinan, 250014 Shandong China; 2 Institute of Poultry Science, Shandong Academy of Agricultural Sciences, 1, JiaoXiao Road, Jinan, 250023 Shandong China; 3 College of Veterinary Medicine, South China Agricultural University, 483, Wushan Road, Guangzhou, 510642 Guangdong China; 4 Department of Veterinary and Biomedical Sciences and Department of Biology and Microbiology, South Dakota State University, Brookings, SD 57007 USA

**Keywords:** H5N1, H9N2, Innate immune-related genes, Real-time quantitative PCR, Subtype-dependent host response

## Abstract

H5N1 and H9N2 viruses are important causes of avian influenza in China. H5N1 is typically associated with severe to fatal disease in poultry, while H9N2 is usually associated with mild disease. Differences in viral virulence prompted us to investigate whether innate immune responses would be differentially regulated following infection by H5N1 and H9N2 viruses. To address this hypothesis, expression of a panel of innate immune-related genes including IFN-α, IFN-β, Mx1, OASL, ISG12, IFIT5, IRF7, USP18, SST, and KHSRP in immortal DF-1 cells following H5N1 and H9N2 infection was analyzed and compared by real-time quantitative RT-PCR. Cells infected by either virus overall exhibited a similar expression profile for four ISGs (Mx1, OASL, ISG12, and IFIT5), IFN-α, IFN-β, and SST gene. However, two immune-regulatory genes (IRF7 and KHSRP) were not responsive to highly pathogenic H5N1 infection but were strongly up-regulated in DF-1 cells infected with low pathogenic H9N2 infection. The subtype-dependent host response observed in this study offers new insights into the potential roles of IRF7 and KHSRP in control and modulation of the replication and virulence of different subtypes or strains of avian influenza A virus.

## Introduction

Avian influenza virus (AIV) can cause influenza in chickens with a spectrum of clinical manifestations, ranging from asymptomatic infection or mild respiratory syndrome to severe and fatal disease. Infection with highly pathogenic AIV (HPAIV) usually leads to systemic “fowl plague” disease with high mortality rates. HPAIV can disseminate to many tissues and organs following infection, including those in the cardiovascular, nervous, respiratory, and urinary systems [1]. However, infection with low pathogenic AIV (LPAIV) is often associated with mild respiratory syndrome. Despite lower virulence, the LPAIV still represents a constant and serious threat to the worldwide poultry industry. AIV infection often results in a virus-induced cytokine deregulation or a “cytokine storm” typically characterized by the presence of elevated levels of pro-inflammatory cytokines and an interferon (IFN) response [2, 3]. Type I IFN response (such as IFN-α and IFN-β) represents the first signaling mechanism to be activated by viral infection, thereby mediating a wide variety of antiviral effects [4, 5]. In mammals, IFN-α/β binds to IFN alpha–beta receptor (IFNAR) on the cell surface and induces an antiviral state characterized by the production of more than 300 IFN-stimulated proteins (ISGs), such as Myxovirus (influenza virus) resistance 1 (Mx1), 2′-5′-oligoadenylate synthetase (OAS), IFN-stimulated gene 15 (ISG15), IFN-stimulated gene 12 (ISG12), interferon-induced protein with tetratricopeptide repeats (IFIT) genes, and interferon response factor 7 (IRF7) [6]. Their antiviral functions have been well documented in mammals recently [7] but are not fully described in chicken cells.

Several avian homologs of ISGs and IFN regulatory factor (IRF) proteins were identified, such as Mx1, OASL, ISG12, IFIT5, and IRF7 [8–12]. Among them, the transcription factor IRF7 plays an important role in the promotion of IFN expression, creating a positive feedback loop [13]. Ubiquitin-specific peptidase 18 (USP18) is an interferon-stimulated gene 15-specific protease, involving in this IFN-mediated antiviral signaling pathway [14–16].

Interactions between virus and the host occur at two stages: the virus’s ability to gain access to the target cell for replication, and the competition between the virus and host cells to control the cellular protein synthesis machinery for their respective benefits. The virus–host interaction is largely determined by the virulence factors of the pathogen and the host immune response [17], and changes in the extent and pattern of host gene expression may be the result of viral replication. Several studies have been conducted using real-time RT-PCR or microarray methods in order to better understand the interplay between HPAIV and avian cells [12, 18–20]. A robust IFN I-associated response was observed in these investigations. To our knowledge, comparative analysis of host gene expressions in response to LPAIV such as H9N2 and HPAIV has not been extensively characterized.

DF-1 is a contiguous cell line of chicken embryo fibroblasts that become spontaneously immortalized without any viral or chemical treatment. They have been widely used in avian virology research including avian influenza viruses because of their susceptibility to virus infection [21, 22]. Here, we employed DF-1 cells to characterize and compare differential host IFN I-associated gene responses upon infection with highly pathogenic H5N1 or low pathogenic H9N2 viruses.

## Materials and methods

### Cell and virus

DF-1 cells were grown in Dulbecco’s modified Eagle’s medium (DMEM) supplemented with 10 % Fetal Bovine Serum (FBS) at 37 °C with 5 % CO_2_. Culture medium was changed every 2 days. Cell passaging was conducted by digestion of cells with 0.25 % trypsin-EDTA and subsequent passage to new flaks at a concentration of 10^5^ cells per mL. High pathogenic avian influenza virus H5N1 virus, A/CK/China/1215/2012, was isolated from a live bird. Low pathogenic avian influenza H9N2 subtype S2 strain (A/chicken/Shandong/2/02) was provided by the Institute of Poultry Science, Shandong Academy of Agricultural Sciences. Viruses were propagated in DF-1 cells at an MOI of 0.01 for 48 h prior to collection for preparation of virus stocks. The medium for H9N2 virus cultivation contained 0.25 µg/mL TPCK-trypsin (Sigma, USA). All infectious materials were handled under the biosafety level 3 (BSL-3) condition, kindly provided by College of Veterinary Medicine, South China Agricultural University.

### Hemagglutination and TCID_50_ assays

The hemagglutination assay was carried out in V-bottom 96-well plates. Serial twofold dilutions of viruses (50 μL) were mixed with an equal volume of a 1 % suspension (v/v) of chicken erythrocytes and incubated at room temperature for 30 min. Wells containing an adherent, homogeneous layer of erythrocytes were scored as positive. The TCID_50_ assay was carried out in a 96-well plate with monolayer DF-1 cells that were infected with 0.1 mL of tenfold series dilutions of viral samples. The medium for cultivation of H9N2 virus contained 0.25 µg/mL TPCK-trypsin. After 4 days of incubation at 37 °C, HA was tested to measure the infectivity ratio for each individual dilution. Then the TCID_50_ of virus was calculated using the standard method of Reed and Muench [23].

### Replication kinetics

Viral replication kinetics experiments were performed on the monolayers of DF-1 cells in 12-well plates (Corning, China). Virus titers were determined by a HA assay and then reported as TCID_50_. Briefly, 1 × 10^6^ cells per well were infected with either virus at MOIs of 1 (2 × 10^6^ TCID_50_ in 0.1 mL), 0.1 (2 × 10^5^ TCID_50_ in 0.1 mL), and 0.01 (2 × 10^4^ TCID_50_ in 0.1 mL), respectively. The medium for cultivation of H9N2 virus contained 0.25 µg/mL TPCK-trypsin. Culture supernatants were collected at 0, 2, 12, 24, 36, 48, 60, and 72 h post-infection (pi), respectively. After centrifugation at 2000 rpm (Sorvall Legend Mach 1.6R, rotor 75003348) for 10 min to remove cellular debris, samples were stored at −80 °C until they were further analyzed for TCID_50_. For every time point, three independent assays were performed for both viruses with each sample analyzed in triplicate.

### RNA extractions from virus-infected cells

Adherent DF-1 cells were passaged 24 h before inoculation. For each well, DF-1 cells were adjusted to 2.0 × 10^6^ per well in 6-well plates (Corning, China) and then infected with H5N1 and H9N2 virus at an MOI of 1 (4 × 10^6^ TCID_50_ in 0.2 mL). After 1 h of incubation at 37 °C and 5 % CO_2_ to allow virus adsorption, cells were washed once with phosphate buffered saline (PBS) and further maintained at 37 °C and 5 % CO_2_ in 2 mL of medium. The medium for H9N2 virus cultivation contained 0.25 µg/mL TPCK-trypsin. Cells were collected at multiple time points following virus infection: 0, 3, 6, 9, 12, and 15 h, and total RNAs were extracted from these samples for real-time RT-PCR experiment. All infectious materials were handled under the biosafety level 3 (BSL-3) condition.

### Real-time RT-PCR

Total RNA was extracted from non-infected and infected cells using an RNeasy Mini Kit (Axygen, China) following the manufacturer’s instructions. Total RNA was converted to cDNA using the ReverTra Ace qPCR RT Master Mix with gDNA Remover (Toyobo, Japan). Real-time PCR was carried out with 2 μL cDNA in a total 25 μL using SYBR Green PCR Master Mix (Toyobo, Japan) on an ABI 7300 Real-Time System (Applied Biosystems, USA) following provided instructions. Primers were designed based on published sequences in NCBI database, and their accession numbers are shown in Table 1. Primer pairs were selected based on the specificity as determined by dissociation curves. PCR conditions were the same for each targeted gene amplification as follows: 95 °C for 40 s, followed by 40 cycles of 95 °C for 10 s, 55 °C for 20 s, and 72 °C for 20 s. An exception was that 53 °C, not 55 °C, was used as an annealing temperature for the amplification of KHSRP. For every gene at each time point, four independent assays were performed with each sample analyzed in triplicate. The PCR products were detected on 1.5 % agarose gel and used directly for sequencing in order to confirm the identities of the genes. The relative expression levels of the target genes were analyzed using the 2^−∆∆Ct^ method [24].

**Table 1:** Primers used in the study

Primer	Sequence (5′-3′)	Product size (bp)	Gene accession no.
Mx1	F: AAgCCTgAgCATgAgCAgAA R: TCTCAggCTgTCAACAAgATCAA	138	NM_204609.1
OASL	F: AgATgTTgAAgCCgAAgTACCC R: CTgAAgTCCTCCCTgCCTgT	106	NM_205041.1
ISG12-2	F: TCAATgggTggCAAAggAg R: TACAgggAgAgCAAAgAAgAgAAgA	129	NM_001001296.5
IFIT5	F: CAgAATTTAATgCCggCTATgC R: TgCAAgTAAAgCCAAAAgATAAgTgT	149	XM_421662.4
IFN-α	F: CAACCTTCACCTCgCCATCA R: TTgTggATgTgCAggAACCAg	129	GU119896.1
IFN-β	F: CCTCAACCAgATCCAgCATT R: ggATgAggCTgTgAgAggAg	259	AY831397
USP18	F: CAACgTgggAAgAggAgAAA R: ACTTCATgAgCggAgAAggA	125	XM_416398.3
SST	F: ggTCCACggTTATggTgAAAg R: ggTCAgAAATCACAACTCAAgCA	118	NM_205336.1
IRF3/7	F: ACTgACCAgCCCAggAACTCT R: AAggCTTTCCCAACCACAAA	70	NM_205372.1
KHSRP	F: CAgCggggAAATgATTAAgAAg R: TTTgTgTgTggggATggAgA	283	NM_204277.1
β-actin	F: ATTgTCCACCgCAAATgCTTC R: AAATAAAgCCATgCCAATCTCgTC	113	NM_205518.1

### Calculations and statistics

The house keeping gene β-actin was used as an internal control, and quantification of the transcripts was performed by the 2^−∆∆Ct^ method. All the primers have been verified using the optimal real-time PCR conditions to ensure target gene and β-actin amplified simultaneously. Each subsequent time point (*t* = 3 h, 6, 9 h, 12 and 15 h hpi) was compared against baseline (*t* = 0 h hpi) transcript level to achieve ∆∆Ct. Logarithmic transformation of 2^−∆∆Ct^ was performed on fold change values using Microsoft Excel 2007. Besides, the logarithmic transformation of 2^−∆Ct^ targets for every gene at different time points was used to conduct statistical analysis of H5N1 or H9N2 transcripts. Standard error was calculated according to the standard method from four replicates of each gene tested. *P* value <0.05, 0.01, and 0.001 was considered statistically significant. Statistical analyses on the data obtained between 0 hpi and subsequent time points were performed using one-way ANOVA of software program SPSS 17.0 (SPSS Inc., Illinois). Two-way ANOVA was employed to perform the statistical analysis on the data obtained between H5N1 and H9N2 viruses for each time point post-infection. All graphs were accomplished using GraphPad Prism 5.

## Results

### Growth kinetics of H5N1 and H9N2 strains with different MOIs

**Fig. 1:**
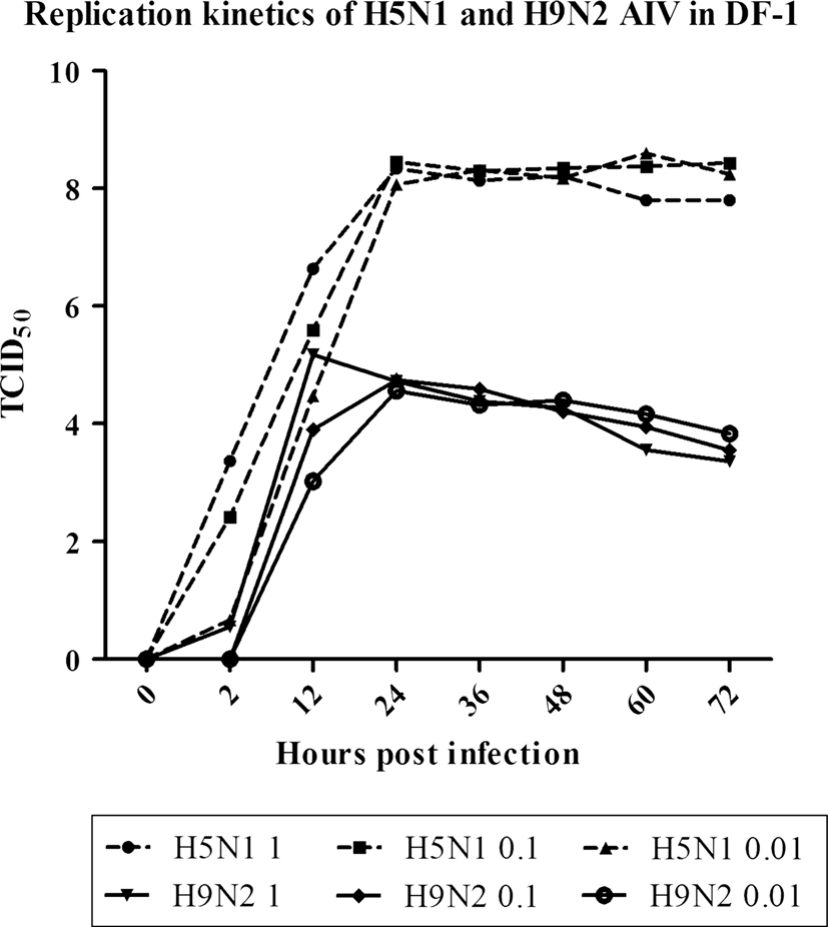
Replication kinetics of H5N1 (1215 strain) and H9N2 (S2 strain) viruses in DF-1 cells. Monolayers of DF-1 cells were infected with MOIs of 1, 0.1, and 0.01, respectively. The medium for H9N2 virus cultivation contained 0.25 µg/mL TPCK-trypsin. Culture supernatants were collected at indicated time points. Virus titers were determined by cytopathic effect (CPE) and reported as tissue culture infectious dose (TCID_50_). *Graphs* represent mean + SEM of three independent experiments, each assayed in triplicate. Replication kinetic curves of H9N2 and H5N1 viruses are indicated by a *solid line* and a *broken line* with *black color*, respectively

The kinetics of replication of H5N1 virus compared with H9N2 virus were measured and compared for infectious titers (TCID_50_) as a function of time. The data in Fig. 1 demonstrated that DF-1 supported the replication of both H5N1 and H9N2 viruses, though the levels of virus production between H5N1 and H9N2 viruses differed significantly over time (*p* value <0.01). H5N1 virus replicated efficiently, reaching up to 10^8.0^ TCID_50_/mL at 24 h post-infection. In contrast, H9N2 virus had a significantly lower virus replication with peak virus titers reaching 10^4.5^ TCID_50_/mL. Interestingly, dose-dependent effects on the level of virus replication for both viruses were discernible during the first 12 h of infection (*p* value <0.01), but the effects were negligible beyond this time point during the 72-h period of replication kinetics experiment.

To determine the ratio of the infectious titer (i.e., TCID_50_) to HA unit for each virus stock used in previous study, we performed HA assays. We found that H5N1 virus had 10^7.33^ TCID_50_ (0.1 mL) with HA titer 2^7^, while H9N2 virus had 10^6.2^ TCID_50_ (0.1 mL) with HA titer 2^6^. The relative ratio of TCID_50_ and HA unit in H5N1 virus was about sevenfold higher than that observed in H9N2 virus. This analysis indicated that H9N2 virus might produce more defective particles than H5N1 virus, which warrants future mechanistic investigation.

### Analysis of differential expression patterns of immune-defensive genes

**Fig. 2:**
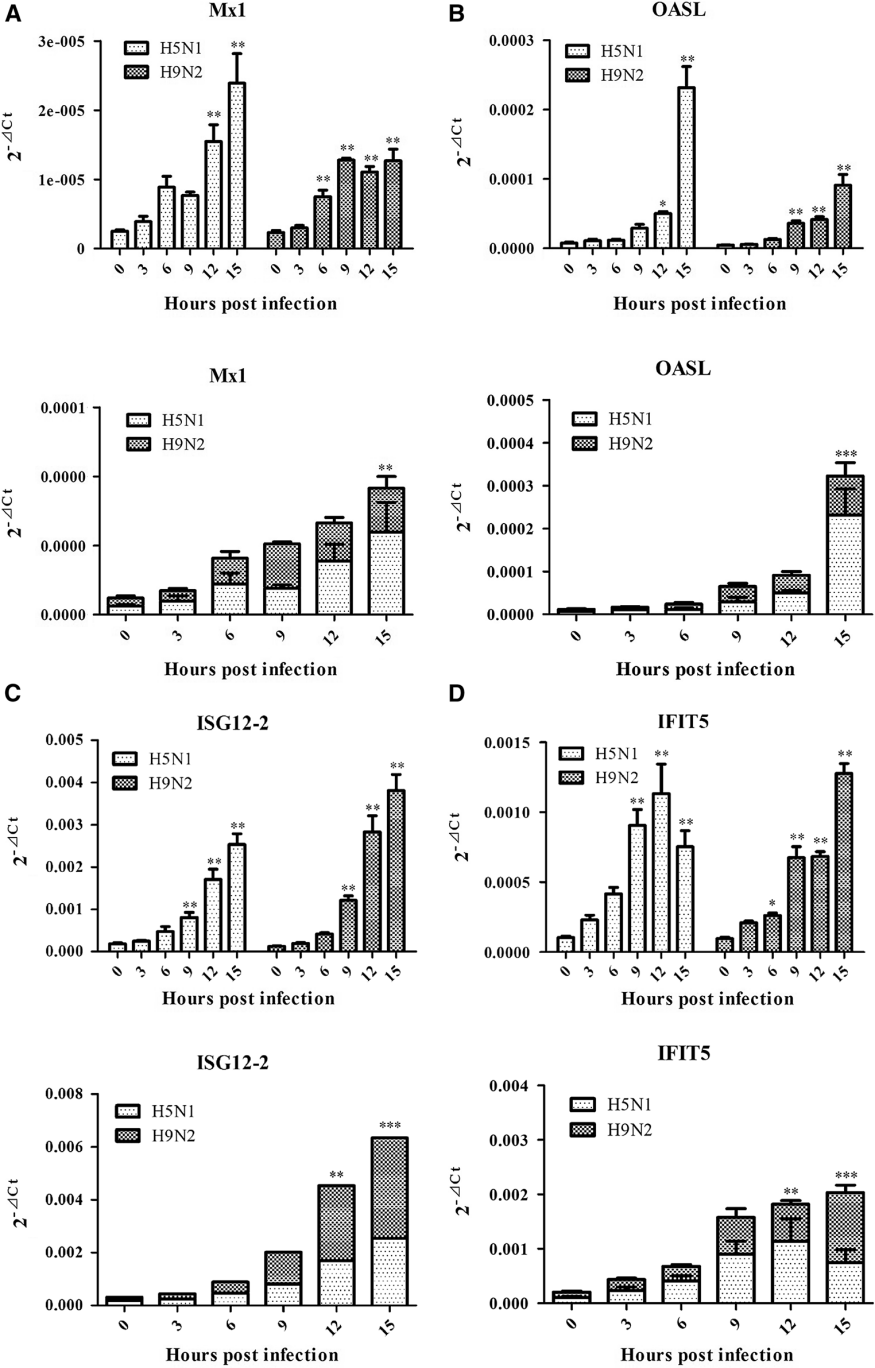

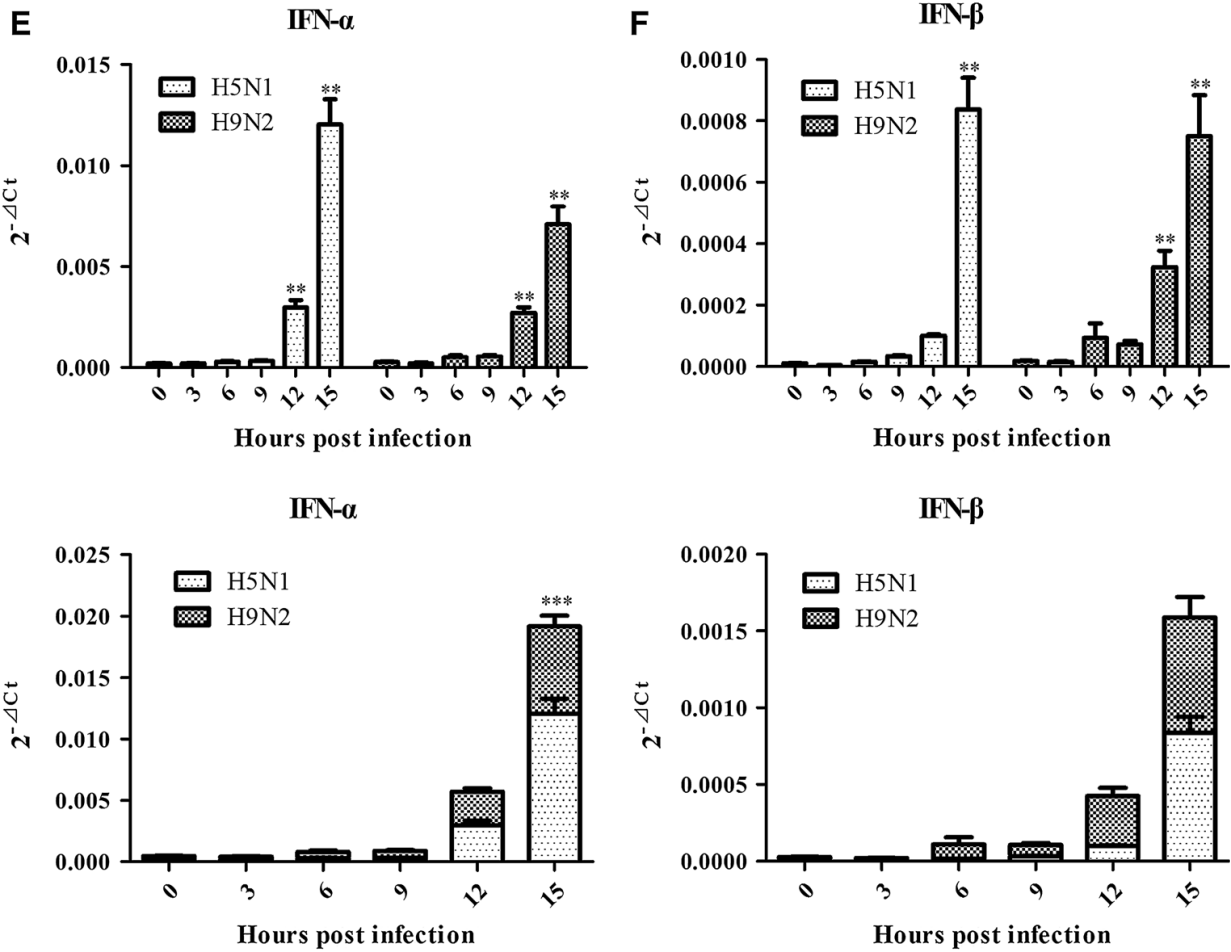
Analysis of differential expression of four immune-defensive genes and IFN-α/β in response to H5N1 and H9N2 infection. The genes tested in this study included Mx1, OASL, ISG12-2, IFIT5, and IFN-α/β. For every target gene, the data were normalized to β-actin mRNA to achieve ∆Ct. The linear data from 2^−∆Ct^ were used for statistical analysis using ANOVA method. The *top* portion of each *panel* indicated the data comparison (statistical analysis with one-way ANOVA method) among different time points following infection within each virus (H5N1 or H9N2), while the *bottom* portion displayed the data comparison between H5N1 and H9N2 viruses on the same time points following infection (statistical analysis with two-way ANOVA method). *Graphs* represent mean + SEM of four independent experiments with each sample analyzed in triplicate, (*), (**), and (***) indicating significant difference

**Fig. 3:**
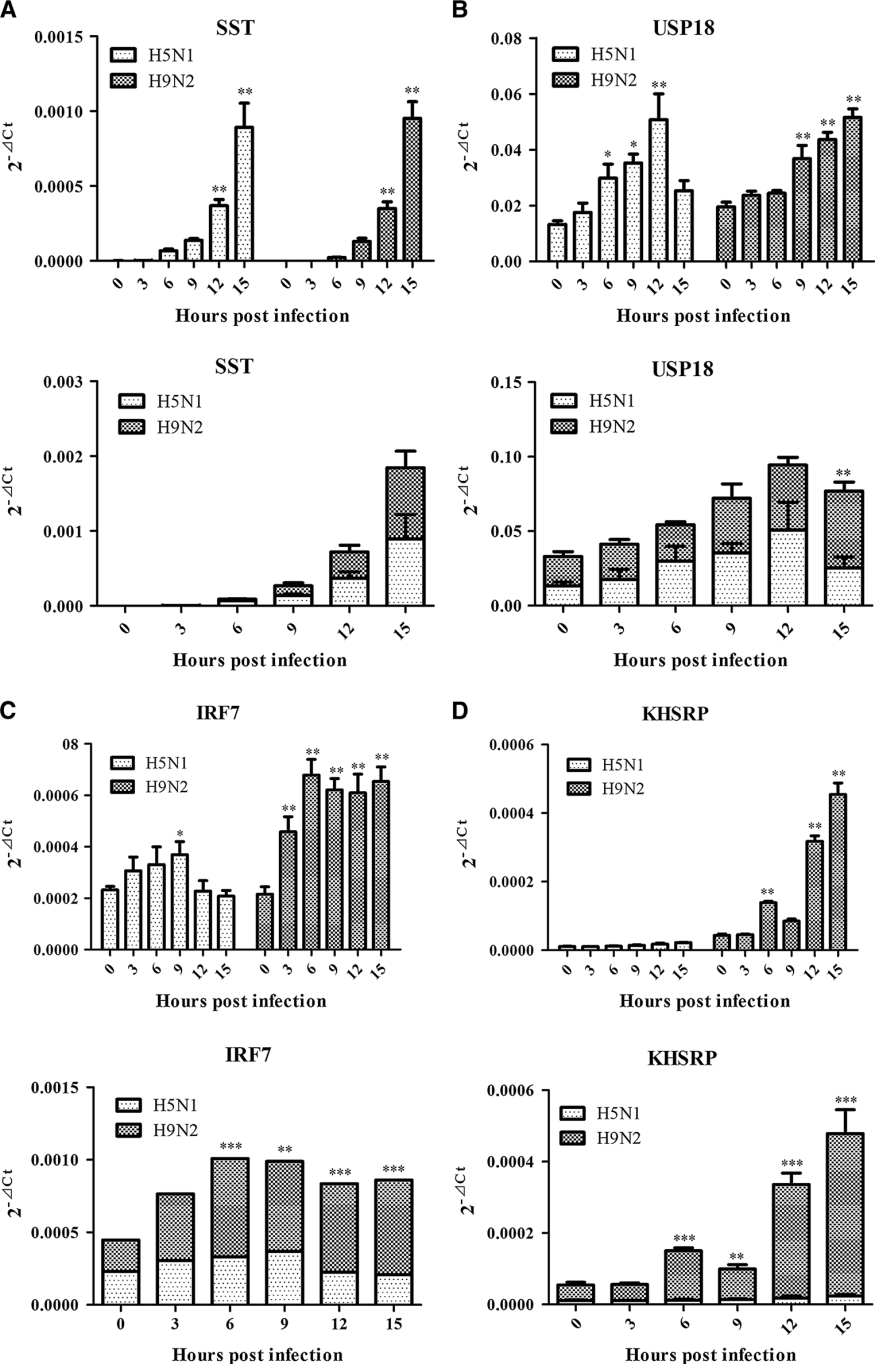
Analysis of differential expression of four immune-regulatory or immune-related genes in response to H5N1 and H9N2 infection. The genes tested in this study included USP18, IRF7, SST, and KHSRP. For every target gene, data were normalized to β-actin mRNA to achieve ∆Ct. The linear data from 2^−∆Ct^ were used for statistical analysis using ANOVA method. The *top* portion of each panel indicated the data comparison (statistical analysis with one-way ANOVA method) among different time points following infection within each virus (H5N1 or H9N2), while the *bottom* portion displayed the data comparison between H5N1 and H9N2 viruses on the same time points following infection (statistical analysis with two-way ANOVA method). *Graphs* represent mean + SEM of four independent experiments with each sample analyzed in triplicate, (*), (**), and (***) indicating significant difference

Temporal analysis of differential expression of six immune-defensive genes discriminated the host responses from both viruses (Fig. 2; Table 2). We used one-way ANOVA method to calculate statistical differences on the data observed among different time points following infection within each virus (H5N1 or H9N2), which was shown in the top portion of each panel representing each individual genes analyzed (Fig. 2). Two-way ANOVA analysis was employed to analyze significant differences on the data observed between H5N1 and H9N2 viruses on the same time points following infection, which was displayed in the bottom portion of each panel (Fig. 2). This approach was also used in Fig. 3 to analyze another set of gene expressions.

**Table 2:** Differential gene expression infected with H5N1 and H9N2 viruses in DF-1

Gene	Time points	H5N1			H9N2		
		Fold changes	Range up	Range down	Fold changes	Range up	Range down
Mx1	0	1.00	1.27	0.79	1.00	1.46	0.69
	3	1.49	1.00	2.22	1.30	1.88	0.90
	6	3.4	2.29	5.05	3.24	4.72	2.22
	9	3.1	2.49	3.78	5.68	7.44	4.33
	12	6	4.11	8.69	4.87	6.63	3.57
	15	9.2	6.13	13.69	5.48	7.99	3.76
OASL	0	1.00	0.6	1.68	1.00	1.49	0.68
	3	1.46	0.87	2.46	1.27	1.78	0.90
	6	1.59	1.00	2.51	2.79	4.12	1.89
	9	3.91	2.40	6.41	8.08	11.34	5.76
	12	7.06	4.86	10.27	9.33	13.12	6.64
	15	32	20.68	49.52	19.87	30.79	12.82
ISG12-2	0	1.00	0.72	1.39	1.00	1.42	0.7
	3	1.34	1.01	1.78	1.59	2.21	1.14
	6	2.32	1.30	4.12	3.48	4.67	2.59
	9	4.24	2.86	6.30	10.06	13.77	7.34
	12	8.97	6.04	13.32	23.18	33.50	16.00
	15	13.5	9.78	18.77	31.56	44.03	22.60
IFIT5	0	1.00	0.74	1.36	1.00	1.33	0.75
	3	2.18	1.50	3.18	2.18	2.75	1.73
	6	4.0	2.93	5.47	2.71	3.45	2.13
	9	8.65	6.12	12.23	6.90	9.29	5.13
	12	10.43	6.42	16.94	7.12	8.91	5.70
	15	7.12	4.87	10.43	13.27	16.7	10.52
IFN-α	0	1.00	1.65	0.60	1.00	1.38	0.73
	3	1.10	1.60	0.75	0.71	1.20	0.42
	6	1.47	2.28	0.95	1.85	2.96	1.16
	9	1.73	2.52	1.18	2.07	2.86	1.50
	12	15.49	23.70	10.12	10.21	13.58	7.67
	15	63.12	94.50	42.16	26.48	36.59	19.16
IFN-β	0	1.00	1.52	0.66	1.00	1.36	0.74
	3	0.47	0.66	0.34	0.88	1.27	0.61
	6	1.49	2.11	1.06	3.99	10.72	1.48
	9	3.27	4.52	2.36	4.01	5.68	2.84
	12	9.75	13.27	7.17	18.09	25.77	12.70
	15	80.47	116.86	55.41	41.78	60.35	28.92

Among these genes analyzed, Mx1, ISG12, and OASL genes had a similar response between DF-1 cells infected with H5N1 and H9N2, respectively. These three genes were significantly upregulated starting at 6 or 9 h post-infection and steadily rising until 15 h post-infection (Fig. 2a–c; Table 2). Analysis of IFIT5 gene expression revealed a slight difference in terms of host response between two viruses. At early time points, IFIT5 gene expression was similar in DF-1 cells infected by both H5N1 and H9N2 viruses. However, at 15 hpi, a decline of IFIT5 gene expression was observed in H5N1 infection (*p* value <0.05, not shown in Fig. 2d). At this time point, H9N2-infected cells exhibited a significant increase of IFIT5 gene expression compared to those expressed at early time points (*p* value <0.05) (Fig. 2d; Table 2). In terms of the differential expression of IFN-α and IFN-β genes, we found that both gene expressions were not induced significantly at three time points (3, 6, and 9 hpi) in virus-infected cells. However, after 9 hpi, a significant up-regulation of IFN-α and IFN-β gene expression was observed in cells infected by both viruses (Fig. 2e, f; Table 2). This result indicated a strong ability of both viruses in suppression of IFN genes expression at the early stage of virus replication.

These results were also generally supported by two-way ANOVA analysis focusing on the differences in host response to H5N1 and H9N2 viruses. H5N1 infection in DF-1 cells induced higher levels of Mx1 and OASL gene expression than H9N2 infection at 15 hpi (*p* value <0.01) (Fig. 2a, b). In terms of ISG12 gene, DF-1 cells infected with H9N2 virus resulted in the level of its expression higher than that in H5N1-infected cells with significant difference at 12 and 15hpi (*p* value <0.01) (Fig. 2c), while IFIT5 gene expression was distinct between two viruses in terms of host response. For example, the expression of IFIT5 gene reached the peak at 12hpi following H5N1 infection and declined significantly at 15 hpi, while at this time point, H9N2 virus infection induced the most abundant expression of IFIT5 gene in DF-1 cells with significant difference compared to its expression in H5N1 virus-infected cells (*p* value <0.001) (Fig. 2d). These ISGs analyzed in this study consisted of Mx1, ISG12, OASL, and IFIT5 that are downstream genes of IFN-α/β. Both viruses induced a similar expression level of IFN-α and IFN-β genes. IFN-β gene displayed similar expression level without significant differences observed between two viruses. However, an exception to above was H5N1 virus that induced higher expression of IFN-α gene at 15 hpi than its counterpart H9N2 virus in infected cells (*p* value <0.001) (Fig. 2e, f)

### Analysis of differential expression patterns of IRF7, USP18, KHSRP, and SST

Analysis of the differential expression dynamics of four immune-regulatory or immune-related genes resulted in more diversified phenotypes in terms of the host responses to two different subtypes of avian influenza virus (Fig. 3; Table 3). A steady increase in the SST gene expression was observed in DF-1 cells infected by both H5N1 and H9N2 viruses, though the differences were not statistically significant between these two viruses (Fig. 3a; Table 3). Similar to IFIT5 expression pattern observed above that could distinguish the two viruses, USP18 gene was also significantly increased until 12 hpi followed by a marked reduction at 15 hpi (*p* value <0.05)which occurred only in H5N1-infected cells. A continuous increase in USP18 gene expression was displayed at various time points following H9N2 virus infection (Fig. 3b; Table 3). Remarkably, no or little changes in the expression patterns of IRF7 and KHSRP were observed in H5N1-infected DF-1 cells. In contrast, a dynamic up-regulation of both genes was present in DF-1 cells infected with H9N2 virus (Fig. 3a, d; Table 3). Further comparison between the two viruses demonstrated that KHSRP and IRF7 genes in H9N2 virus-infected DF-1 cells displayed significantly high expressions at 6, 9, 12, and 15 hpi, respectively, than their expression levels in H5N1-infected cells (Fig. 3c, d). This is an interesting finding in that the measurement of these two genes can distinguish H5N1 and H9N2 viruses in terms of the host response.

**Table 3:** Differential gene expression infected with H5N1 and H9N2 viruses in DF-1

Gene	Time points	H5N1			H9N2		
		Fold changes	Range up	Range down	Fold changes	Range up	Range down
SST	0	1.00	0.85	1.18	1.00	1.44	0.69
	3	1.17	0.80	1.69	1.16	1.70	0.79
	6	52.98	37.73	74.41	24.85	33.87	18.23
	9	112.21	92.41	136.24	143.51	215.44	95.59
	12	299.99	235.36	382.35	388.70	575.70	262.44
	15	706.11	495.85	1005.54	1073.00	1499.78	767.73
USP18	0	1.00	0.76	1.32	1.00	1.27	0.79
	3	1.25	0.79	1.98	1.21	1.49	0.98
	6	2.18	1.49	3.19	1.26	1.51	1.05
	9	2.65	2.07	3.40	1.85	2.49	1.38
	12	3.63	2.27	5.82	2.24	2.75	1.82
	15	1.87	1.34	2.61	2.65	3.25	2.16
KHSRP	0	1.00	0.74	1.36	1.00	1.31	0.77
	3	0.99	0.75	1.29	1.05	1.30	0.85
	6	1.05	0.74	1.49	3.23	3.93	2.66
	9	1.34	1.02	1.76	1.96	2.50	1.54
	12	1.55	0.99	2.45	7.39	9.15	5.96
	15	2.27	1.73	2.97	10.50	13.39	8.23
IRF7	0	1.00	0.85	1.18	1.00	1.45	0.69
	3	1.27	0.86	1.85	2.13	3.12	1.45
	6	1.34	0.86	2.08	3.19	4.43	2.29
	9	1.55	1.16	2.08	2.94	3.95	2.19
	12	0.93	0.62	1.4	2.85	4.03	2.01
	15	0.88	0.69	1.13	3.08	4.22	2.25

## Discussion

Innate immunity provides a first line of defense against pathogens and can be rapidly activated following viral infection. Activation or response of ISGs might be different against various pathogens or different strains of the same pathogen such as influenza A virus. Using quantitative RT-PCR for the analysis of a panel of innate immunity-related genes, we observed some interesting antiviral responses that can discriminate highly pathogenic H5N1 from low pathogenic H9N2 viruses in infected DF-1 cells. In this study, mRNA samples collected at multiple time points following virus infection were examined and compared for the differential host gene responses to H5N1 and H9N2 viruses with a primary focus on a panel of immune-defensive and immune-regulatory genes. Samples from later time points beyond 15 h were not selected in our study because infected DF-1 cells appeared cytopathic effects (CPE) (data not shown).

IFN-stimulated effector genes including Mx1, OASL, ISG12-2, and IFIT5 were selected in this study because these genes are well characterized in the context of influenza virus infection. IFN-α and IFN-β were also included because they are upstream genes of these ISGs analyzed and because of their ability to trigger a cascade of ISG response that can directly interfere with influenza virus replication. We also selected two immune-regulatory genes, IRF7 and USP18, because of their roles in the modulation of antiviral signaling pathways [13–16]. Two additional genes included were K-homology splicing regulatory protein (KHSRP) and Somatostatin (SST) genes. These two genes are believed to function as multifunctional RNA-binding protein [25] and play roles in growth, digestion (metabolism), and reproduction [26–29]. Inclusion of these two genes in our analysis is due to the related evidence showing that KHSRP had some roles in the regulation of NF-κB and the JAK2-STAT-1a pathways [30–33], while SST functions in control growth, metabolism, and reproduction of chicken [34], which are potentially associated with influenza virus infection. In addition, selection of DF-1 cells in this project was based on the fact that it is susceptible to infection by both H5N1 and H9N2 subtypes of AIV [21, 22].

In this study, either virus induced a significant expression of IFN-α and IFN-β genes at 12 hpi or 15 hpi. It was reported that influenza A could antagonist IFN-α/β induction in infected mammalian cells and avian cells via the NS1 protein [35, 36], which may explain the delayed induction of IFN-β. This observation coincided with the appearance of CPE at 15 hpi and later time points in DF-1 cells, suggesting that either virus has disrupted the innate immunity defense and subsequently established virus infection.

A particular intriguing observation is that H9N2 virus infection triggered a dynamic up-regulation of both KHSRP and IRF-7, but their expressions were not altered in cells infected by H5N1 virus. The observed differential gene expression between the two viruses could not attribute to the different levels of virus replication because similar expression cascades for Mx1, ISG12, SST, and OASL genes were observed between H5N1- and H9N2-infected cells. Expression of KHSRP was not responsive to H5N1 infection but had a strong induction after H9N2 infection, suggesting that it plays a potential role in the replication and pathogenesis of low pathogenic H9N2 virus infection. KHSRP played some roles in p38MAPK, NF-κB, and JAK2-STAT-1a pathways, and these signaling pathways were reported to be in association with host defense of avian influenza virus infection in avian-origin cells [30–33, 37, 38]. Induction of KHSRP gene expression may reflect a feedback loop for which KHSRP stimulates IFN-α/β secretion which in turn triggers downstream expression of ISGs.

The similar expression pattern occurred in IRF7 gene where its expression was significantly up-regulated in DF-1 cells infected by H9N2, not H5N1. IRF7 constitutes a part of a positive feedback loop leading to the amplification of IFN gene expression. Activated IRF7 cooperates with IRF3 and stimulates expression of the numerous IFN-related genes leading to a broad IFN-α response in mammals. Interestingly, the chicken genome does not encode IRF3 gene [39, 40]. We speculated that IRF7 alone in chicken and other avian species might fulfill dual functions of IRF3/IRF7 in mammals toward the induction of IFN-α/β response. H5N1 virus infection induced little response of IRF7 gene. In contrast, infection with H9N2 virus resulted in a robust response of IRF7 in infected DF-1 cells. Similar to possible subtype-specific KHSRP gene response, we hypothesize that IRF7 plays an important role in the replication and pathogenesis of low pathogenic H9N2 virus, which will be addressed in a future study.

The other interesting observation that can distinguish two viruses includes IFIT5 and USP18 gene expression patterns (Figs. 2d, 3b; Tables 2, 3). There was a continuous upward increase for IFIT5 and USP18 genes until 12 hpi followed by a significant decline at 15 hpi in H5N1 virus infection. In contrast, a steady rise in both gene expressions at various time points was observed in DF-1 cells infected by H9N2 virus. Influence of these differential gene expressions on viral pathogenesis needs to be further investigated.

In summary, comparative analysis of innate immune responses against high (H5N1) and low (H9N2) pathogenic avian influenza A viruses in DF-1 cells revealed that the responses of several selected genes (IFN-α, IFN-β, ISG12, OASL, and SST) were similar between two viruses. However, host responses to H5N1 and H9N2 viruses were markedly different in two immune-regulatory genes (KHSRP and IRF7). Our study showed a strong response to H9N2 virus infection and no or little response to H5N1 virus infection. Results of our experiments shall provide new information about the role of differential regulation of innate immune response in modulation of viral virulence and replication of different subtypes or strains of avian influenza virus.
